# Correction: African American mitochondrial DNAs often match mtDNAs found in multiple African ethnic groups

**DOI:** 10.1186/1741-7007-5-13

**Published:** 2007-03-26

**Authors:** Bert Ely, Jamie Lee Wilson, Fatimah Jackson, Bruce A Jackson

**Affiliations:** 1 Department of Biological Sciences, University of South Carolina, Columbia, South Carolina, 29208, USA; 2 Biomedical Engineering and Biotechnology Program, University of Massachusetts, Lowell, MA 01854, USA; 3 Department of Anthropology, University of Maryland, College Park, MD 20742, USA

After the publication of this work [1], we became aware that AFDIL data set used to construct our database of sub-Saharan mtDNA sequences had been mislabelled, and in fact, did not contain Sierra Leone mtDNA sequences. We have obtained the correct Sierra Leone data set from AFDIL, reconstructed the database using the new file, and reanalyzed all of the data. The size of our database was reduced from 3725 to 3717 since the new Sierra Leone data set contained 109 sequences instead of 117 in the mislabelled data set. The swapping of data sets resulted in a cascade of minor corrections to Tables 2, 3, 4, 5, and 6 (see below). We also corrected an error in the entry for BAM013 in Table 7. However, the only major change was that there were a number of matches of African-American sequences to sequences in the mislabelled data set. Thus, the number of African-American matches to single ethnic groups dropped from a total of 16 to 9 (Table 3) representing just 5% of the African American sequences that we compared to the database.

## Figures and Tables

**Table 2:** Characteristics of the sub-Saharan mtDNA HVS-I database

**Region**	**Region matched (%)**							
	Total	Unique^a^	Multiple^b^	West	West Central	South	Southeast	East

West	1520	35	18	23	20	0	1	2
West Central	995	35	28	16	14	0	5	2
South	127	39	15	0	0	39	7	0
Southeast	416	25	54	2	17	1	1	0
East	659	52	14	1	2	0	0	30
Total	3717	37	24					

^a^Haplotypes found once or in a single ethnic group.

^b^Haplotypes found in ethnic groups from three or more regions.

**Table 3:** Number of Perfect Matches to African-American HVS-I sequences

	Sample	
Number of Matched Ethnic Groups	Gullah/Geechee	AFDIL

None	30	43
1	5	4
2–3	6	5
4–9	10	15
>9	23	30
Totals	74	97

**Table 4:** Imperfect matches to the Gullah/Geechee and AFDIL African-American HVS-I sequences

	Number of sequences	Number of ethnic groups matched	Number of sequences
One mismatch	47	1	14
		2–3	5
		4–9	16
		>9	12
More than 1 mismatch	26		ND

**Table 5:** Geographical source of mtDNA HVS-I matches

Number of matches	Gullah/Geechee individuals			AFDIL African American individuals		
	W. Africa	E. Africa	Both	W. Africa	E. Africa	Both

1–5	13	1	2	11	1	2
>5	4	0	24	5	0	33

**Table 6:** Distribution of single region matches

Sample	West	W. Central	East
Gullah/Geechee	7	5	1
AFDIL AA	2	3	1

**Table 7:** Mitochondrial DNA HVS-I sequences included in this study

**Ethnic Group**	**Country**	**Sample Size**	**Reference**
**West Africa:**			

multiple	Senegal	50	Rando et al. 1998 [9]
Serer	Senegal	23	Rando et al. 1998 [9]
Wolof	Senegal	48	Rando et al. 1998 [9]
Mandenka	Senegal	110	Graven et al. 1995 [10] Watson et al. 1997 [11]
multiple	Guiné-Bissau	372	Rosa et al. 2004 [12]
Malinke	Mali	61	Ely et al., unpublished
Bambara	Mali	19	Ely et al., unpublished
Limba	Sierra Leone	67	Jackson et al. 2005 [3]
Loko	Sierra Leone	29	Jackson et al. 2005 [3]
Temne	Sierra Leone	121	Jackson et al. 2005 [3]
Mende	Sierra Leone	59	Jackson et al. 2005 [3]
unknown	Sierra Leone	108	Monson et al. 2002 [13]
Fulbe	Nigeria, Niger	60	Watson et al. 1997 [11]
Hausa	Nigeria, Niger	20	Watson et al. 1997 [11]
Kanuri	Nigeria, Niger	14	Watson et al. 1997 [11]
Songhai	Nigeria, Niger	10	Watson et al. 1997 [11]
Tuareg	Nigeria, Niger	23	Watson et al. 1997 [11]
Yoruba	Nigeria	33	Vigilant et al. 1991 [14]; Watson et al. 1997 [11]
unknown	Cabo Verde	292	Brehm et al. 2002 [15]

**Total**		**1520**	

**West Central Africa:**			

Kotoko	Cameroon	18	Černý et al. 2004 [16]
Hide	Cameroon	23	Černý et al. 2004 [16]
Masa	Cameroon	31	Černý et al. 2004 [16]
Mafa	Cameroon	32	Černý et al. 2004 [16]
Bakaka	Cameroon	50	Coia et al. 2005 [17]
Bamileke	Cameroon	48	Coia et al. 2005 [17]
Bassa	Cameroon	46	Coia et al. 2005 [17]
Daba	Cameroon	20	Coia et al. 2005 [17]
Ewondo	Cameroon	53	Coia et al. 2005 [17]
Fali	Cameroon	41	Coia et al. 2005 [17]
Fulbe	Cameroon	34	Coia et al. 2005 [17]
Mandara	Cameroon	37	Coia et al. 2005 [17]
Podokwo	Cameroon	39	Coia et al. 2005 [17]
Tali	Cameroon	20	Coia et al. 2005 [17]
Tupuri	Cameroon	25	Coia et al. 2005 [17]
Uldeme	Cameroon	28	Coia et al. 2005 [17]
Biaka	Central African Republic	17	Vigilant et al. 1991 [14] Watson et al. 1997 [11]
Mbenzele-Pygmy	Central African Republic	57	Destro-Bisol et al. 2004 [18]
Angolares	São Tomé and Príncipe	30	Trovoada et al. 2004 [19]
Forros	São Tomé and Príncipe	35	Trovoada et al. 2004 [19]
Tongas	São Tomé and Príncipe	38	Trovoada et al. 2004 [19]
unknown	São Tomé and Príncipe	50	Mateu et al. 1997 [20]
Bubi	Equatorial Guinea	45	Mateu et al. 1997 [20]
Fang	Equatorial Guinea	11	Pinto et al. 1996 [21]
Mbuti	Democratic Republic of Congo	13	Vigilant et al. 1991 [14] Watson et al. 1997 [11]
Bantu-speaking	Cabinda	110	Beleza et al. 2005 [4]
Mbundu	Angola	44	Plaza et al. 2004 [22]

**Total**		**995**	

**East Africa:**			

Nuer	South Sudan	11	Krings et al. 1999 [23]
Dinka	South Sudan	47	Krings et al. 1999 [23]
Shilluk	South Sudan	7	Krings et al. 1999 [23]
multiple	Ethiopia	21	Kivisild et al. 2004 [24]
Tigrais	Ethiopia, Eritrea	53	Kivisild et al. 2004 [24]
Gurage	Ethiopia	21	Kivisild et al. 2004 [24]
Afar	Ethiopia	16	Kivisild et al. 2004 [24]
Amhara	Ethiopia	120	Kivisild et al. 2004 [24]
Amhara	Ethiopia	7	Quintana-Murci et al. 1999 [25]
Oromo	Ethiopia	33	Kivisild et al. 2004 [24]
Oromo	Kenya, Ethiopia	18	Quintana-Murci et al. 1999 [25]
unknown	Kenya	100	Brandstätter et al. 2004 [26]
Kikuyu	Kenya	24	Watson et al. 1997 [11]
Turkana	Kenya	37	Watson et al. 1997 [11]
Somali	Kenya, Somalia, Ethiopia	27	Watson et al. 1997 [11]
Hadza	Tanzania	17	Vigilant et al. 1991 [14]
Hadza	Tanzania	49	Knight et al. 2003 [27]
Dakota	Tanzania	18	Knight et al. 2003 [27]
Iraqw	Tanzania	12	Knight et al. 2003 [27]
Sukuma	Tanzania	21	Knight et al. 2003 [27]

**Total**		**659**	

**Southeast Africa:**			

mulitple	Mozambique	109	Pereira et al. 2001 [6]
multiple	Mozambique	307	Salas et al. 2002 [5]

**Total**		**416**	

**South Africa:**			

!Kung	Botswana	34	Vigilant et al. 1991 [14]
!Kung	South Africa	43	Chen et al. 2000 [28]
Khwe	South Africa	31	Chen et al. 2000 [28]
Herero	Bostwana, Namibia	19	Vigilant et al. 1991 [14]

**Total**		**127**	

**Table 8:** Malinke and Bambara Mitochondrial DNA HVS-I sequences included in this study

**ID**	**Ethnicity**	**Haplogroup**	**Hvs-I polymorphisms**^a^
BAM676	Bambara	L1b	126 187 189 223 264 270 278 311
BAM612	Bambara	L1b1	126 187 189 223 256 264 270 278 293 311
BAM595	Bambara	L1b1	126 187 189 223 264 266 270 278 293 311
BAM599	Bambara	L1b1	126 187 189 223 264 266 270 278 293 311
BAM600-2	Bambara	L1b1	126 187 189 223 264 270 278 293 311
BAM060	Bambara	L2a	223 278 294 368 390
BAM598	Bambara	L2a1	189 192 209 223 278 294 309 390
BAM604	Bambara	L2a1a	223 278 286 294 309 390
BAM627	Bambara	L2b	114A 213 223 278 290 355 390
BAM659	Bambara	L2b1	114A 129 213 223 278 362 390
BAM037	Bambara	L2c	129 223 261 278 390
BAM685	Bambara	L2c2	183 223 264 278 320 390
BAM679-1	Bambara	L2c2	223 264 278 390
BAM629	Bambara	L2d2	111A 145 184 223 239 278 292 355 390 399 400
BAM068	Bambara	L3b	124 223 278 362
BAM072	Bambara	L3e2	223 284 320
BAM605	Bambara	L3e3	093 148 223 265 311
BAM027	Bambara	L3f1	049 129 209 223 292 295 311
BAM614	Bambara	L3f1	223 272 292 311
BAM 552	Malinke	L1b	111 126 187 189 223 239 270 278 311
BAM 237	Malinke	L1b	126 187 189 223 239 264 270 278 311
BAM 357	Malinke	L1b	126 187 189 223 239 264 270 278 311
BAM 040	Malinke	L1b	126 187 189 223 264 270 278 311
BAM 385	Malinke	L1b1	093 126 145 187 189 223 264 270 278 293 311
BAM 555	Malinke	L1b1	126 187 189 213 223 260 264 270 278 293 311
BAM 225	Malinke	L1b1	126 187 189 223 264 270 278 293 311 362 400
BAM 407	Malinke	L1c	129 189 215 223 278 294 311 360
BAM 013	Malinke	L2a	189 223 278 390
BAM 397	Malinke	L2a	189 192 223 278 294 390
BAM 221	Malinke	L2a	189 223 278 294 390
BAM 426	Malinke	L2a	223 278 286 294 390
BAM 083	Malinke	L2a	223 278 294 390
BAM 414	Malinke	L2a1	093 189 192 223 265 278 294 309 390
BAM 143	Malinke	L2a1	086 223 230 278 294 309 390
BAM 117	Malinke	L2a1	092 223 278 294 309 390
BAM 341	Malinke	L2a1	093 223 278 294 309 390
BAM 534	Malinke	L2a1	140 189 192 223 278 294 309 390
BAM 665	Malinke	L2a1	189 192 223 266 278 294 309 390
BAM 082	Malinke	L2a1	189 192 223 278 294 309
BAM 174	Malinke	L2a1	192 223 278 294 309 390
BAM 195	Malinke	L2a1	192 223 278 294 309 390
BAM 395	Malinke	L2a1	223 278 294 309 368 390
BAM 406	Malinke	L2a1	223 278 294 309 390
BAM 204	Malinke	L2a1	223 278 309 390
BAM 296	Malinke	L2b1	056 114A 129 213 223 278 362 390
BAM 085	Malinke	L2b1	093 114A 129 213 223 278 355 362 390
BAM 577	Malinke	L2b1	114A 129 213 223 278 311 355 362 390
BAM 290	Malinke	L2b1	114A 129 213 223 278 362 390
BAM 319	Malinke	L2b1	114A 129 213 223 278 362 390
BAM 401	Malinke	L2c	129 223 261 278 362 390
